# Exercise cardiovascular magnetic resonance: development, current utility and future applications

**DOI:** 10.1186/s12968-020-00652-w

**Published:** 2020-09-10

**Authors:** Thomas P. Craven, Connie W. Tsao, Andre La Gerche, Orlando P. Simonetti, John P. Greenwood

**Affiliations:** 1 Leeds Institute of Cardiovascular and Metabolic Medicine, University of Leeds, Leeds, UK; 2 Cardiovascular Division, Beth Israel Deaconess Medical Center, 330 Brookline Ave, RW-453, Boston, MA 02215 USA; 3 Clinical Research Domain, Baker Heart and Diabetes Institute, Melbourne, Australia; 4 National Centre for Sports Cardiology, St Vincent’s Hospital, Fitzroy, Australia; 5 The Ohio State University, Columbus, OH USA

**Keywords:** Cardiovascular magnetic resonance, Exercise cardiovascular magnetic resonance, Stress cardiovascular magnetic resonance, Exercise stress, Treadmill cardiovascular magnetic resonance, Supine cycle ergometer

## Abstract

Stress cardiac imaging is the current first line investigation for coronary artery disease diagnosis and decision making and an adjunctive tool in a range of non-ischaemic cardiovascular diseases. Exercise cardiovascular magnetic resonance (Ex-CMR) has developed over the past 25 years to combine the superior image qualities of CMR with the preferred method of exercise stress. Presently, numerous exercise methods exist, from performing stress on an adjacent CMR compatible treadmill to in-scanner exercise, most commonly on a supine cycle ergometer. Cardiac conditions studied by Ex-CMR are broad, commonly investigating ischaemic heart disease and congenital heart disease but extending to pulmonary hypertension and diabetic heart disease. This review presents an in-depth assessment of the various Ex-CMR stress methods and the varied pulse sequence approaches, including those specially designed for Ex-CMR. Current and future developments in image acquisition are highlighted, and will likely lead to a much greater clinical use of Ex-CMR across a range of cardiovascular conditions.

## Background

Stress testing can be a pivotal tool for the diagnostic and prognostic assessment of cardiovascular disease. Historically for coronary artery disease (CAD), treadmill electocardiography (ECG) was the reference standard [1, 2]. However, the use of stress cardiac imaging for exercise testing has significantly improved the diagnostic accuracy for CAD detection compared to exercise ECG alone [3–8]. Thus stress imaging is now the preferred investigation for CAD diagnosis in intermediate risk patients and a useful tool for prognostication and decision making [9, 10]. Cardiovascular magnetic resonance (CMR) has several well established benefits over alternative imaging modalities, allowing a non-invasive comprehensive multi-parametric assessment, with few limitations from body habitus, no ionizing radiation [11], and is the non-invasive gold standard for bi-ventricular volume and functional assessment [12, 13]. Pharmacological stress CMR has become widely utilised clinically, demonstrating superiority over myocardial perfusion scintigraphy by single photon emission computed tomography (MPS-SPECT) in the diagnosis [14, 15] and prognosis of CAD [16] and recently, a lower incidence of revascularization and non-inferiority in major adverse cardiac events compared to CAD management guided by coronary angiography with fractional flow reserve [17]. However, pharmacological stress has more adverse events than exercise stress, as demonstrated in stress echocardiography [18, 19], contraindications and side effects patients may not tolerate [20] and does not replicate the neurohormonal and haemodynamic changes associated with physical exercise. As such, current guidelines advise physical exercise as the preferred method for stress imaging, when feasible [21, 22]. Exercise imaging studies primarily focus on CAD, however exercise testing is an important decision making tool in numerous cardiac diseases including valvular heart disease [23] and congenital heart disease [24].

Despite the advantages of CMR as a modality and physical exercise advised first line, exercise CMR (Ex-CMR) is not widely used clinically. Limitations include difficulty with image acquisition and quality, the expense of commercially available CMR compatible exercise devices [25] and that exercise testing is technically more difficult than administering pharmacological stress [26]. This review will focus on the recent development of Ex-CMR as a technique, its current utility and challenges, and its potential future applications and technical developments.

## Exercise CMR – methodology and development

Ex-CMR is performed either by exercising outside the scanner bore on a CMR compatible adjacent treadmill [27] or by exercising in the CMR scanner, most commonly using a supine cycle ergometer. Exercising in the CMR environment has safety implications, as patients require rapid removal from the magnet bore and scanner room to facilitate resuscitation in the event of cardiac arrest [28]. Exercise on a CMR compatible adjacent treadmill, utilising a Bruce protocol treadmill test, benefits from the safety of 12-lead ECG monitoring, essential for identifying ECG changes which may prompt test termination, but with the limitation of requiring rapid transfer to the CMR isocenter for post stress imaging. In-scanner Ex-CMR overcomes this limitation, as exercise can be performed in the scanner bore, with imaging performed during exercise or a brief cessation of exercise. However, CMR scanning during exercise creates several issues including increased physical and respiratory motion creating artefacts, ECG gating issues and cannot be monitored by 12-lead ECG [29]. Indeed accurate ST segment monitoring is not feasible within the CMR scanner bore due to the magnetohydrodynamic effect distorting the surface ECG [30]. ECG gating issues can occur at maximal heart rates and during exercise. At maximal heart rates this can be overcome with real time imaging after exercise cessation, as utilised in treadmill Ex-CMR [31], or with ungated real time imaging during maximal supine bicycle exercise [29]. Exercise inherently causes movement, which can result in image acquisition away from the initial planned slice location. Bulk movement can be reduced by using straps around the chest and anterior coil and by counselling/training the patient. However, meticulous image planning is essential to ensure appropriate stress slice localisation. Short axis cine imaging, for ventricular volumetric analysis, should be planned with sufficient slices beyond the base and apex, to account for movement. Repeating left ventricular (LV) and right ventricular (RV) outflow tract views after/during exercise, with free breathing imaging, immediately prior to phase contrast imaging of the aorta or main pulmonary artery allows re-planning to account for movement that may have occurred whilst performing in-scanner exercise. Respiratory navigation can be performed to accommodate for respiratory motion and can be performed retrospectively with ungated real time CMR imaging by manually ‘gating’ respiration using a plethmysograph trace [29].

Numerous exercise CMR studies have been performed using varying methods, including treadmill exercise, supine cycle ergometer or supine stepper-stress, upright cycle ergometry in an open magnet, isometric handgrip exercise (IHG) and prone exercise using either knee flexion or extension with resistance from cables or non-ferromagnetic weights. Similar to exercise echocardiography, the range of applications of Ex-CMR extends beyond CAD to a wide range of cardiac conditions. Each exercise method has inherent benefits and weakness (Table 1). Treadmill exercise, to date, has demonstrated the most clinical utility, being the only validated method for ischaemia detection, however, in-scanner supine cycle ergometer exercise has numerous publications in a broader range of cardiac conditions. Each exercise method will be reviewed including its benefits, limitations, published applications and the technological and imaging sequence developments that have occurred to overcome the described issues of performing Ex-CMR.

**Table 1:** Characteristics and benefits of the varying exercise modalities used in exercise CMR

Exercise type	Treadmill	Upright cycle ergometer	Supine Cycle ergometer	Supine stepper ergometer	Prone exercise	Isometric Handgrip
	Outside MR scanner	Inside MR scanner				
	Dynamic	Dynamic	Dynamic	Dynamic	Dynamic	Static
Common applications	Ischaemia testing (Regional wall motion & perfusion)	Aortic/Pulmonary Flow	Ventricular volumes Aortic/pulmonary flow	Ventricular volumes Aortic/pulmonary flow	Spectroscopy	Spectroscopy Coronary endothelial function
Max exercise intensity^a^	Maximal	Light	Maximal	Submaximal/ Vigorous	Light-Moderate	Very-light
Benefits	-Patients more readily achieve maximal intensity exercise -Diagnostic 12 lead ECG performed during exercise -Treadmill test provides separate prognostic data - Maximal oxygen uptake during exercise on CMR adjacent treadmill feasible -Most natural and tolerated form of exercise	Allows imaging during exercise				
		Allows imaging at multpile exercise levels				
		-Only modality with upright in-scanner exercise -Less claustrophobia in open magnet scanner	-Can be performed to maximal exercise intensity in MR bore.	-Less leg restriction than cycle ergometer		-Stable stress heart rate -Minimal movement -No magnet bore restriction
Weaknesses	- Post stress imaging allows heart rate recovery before imaging - Logistically difficult to image at multiple exercise intensities	Unable to perform 12 lead ECG or accurate ST segment monitoring during in-scanner exercise				
		-Uses open magnet scanner – low field strength (low SNR), limited availability, CMR feasible but non-standard. -Only published in minimal studies to light intensity exercise.	-Cycling can be restricted by magnet bore diameter	- Lower intensity exercise than cycle ergometer	- Uncomfortable form of exercise - Modest exercise feasible - Logistically difficult to increase resistance	-Atypical form of exercise - Limited increase in heart rate

^a^Highest exercise intensity achieved in published research, with intensities defined by American College of sports medicines guidelines [32]. *SNR* = signal-to-noise ratio

### Treadmill exercise CMR

**Fig. 1:**
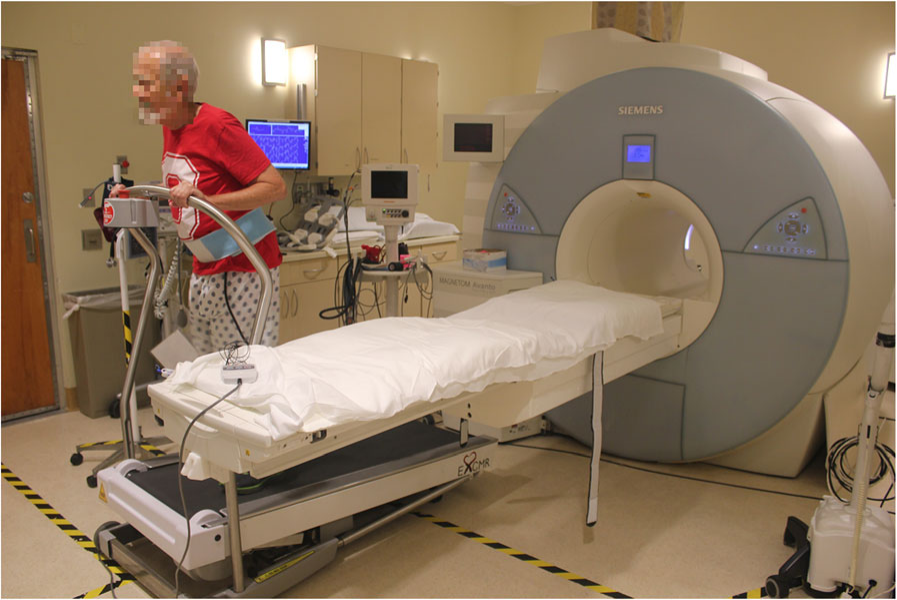
Cardiovascular magnetic resonance (CMR) compatible scanner adjacent treadmill, developed and utilised in ischaemia studies by the Ohio State University Research group [27], reduces transfer times for post stress CMR imaging, whilst still allowing a diagnostic 12-lead ECG treadmill test and a simultaneous maximal oxygen uptake test, if required

Directly analogous to treadmill stress echocardiography, treadmill Ex-CMR is performed to achieve the required exercise intensity/ target heart rate (THR). The patient is then rapidly transferred into the CMR scanner for post stress imaging. Treadmill Ex-CMR has progressed from exercising outside the scanner room [33], to the development of a CMR compatible treadmill to allow exercise to take place inside the scanner room [34] and eventually performed adjacent to the CMR scanner [27] (Fig. 1). For ischaemia studies, this progression has reduced the ‘cooling off period’ from peak stress to image acquisition, as even a 60–90 s delay in performing stress echocardiography image acquisition, has demonstrated recovery of ischaemic regional wall abnormalities and thus decreases the sensitivity of ischaemia detection [35, 36]. Numerous studies in exercise echocardiography have demonstrated the differences between peak and post stress imaging, specifically demonstrating that peak stress imaging has superior sensitivity and accuracy at detecting ischaemia than post stress imaging [35, 37–40]. A direct ‘head-to-head’ comparison in stress echocardiography demonstrated that peak stress supine bicycle echocardiography was superior to post stress treadmill echocardiography in ischaemia detection [41]. Consequently, stress echocardiography guidelines recommend post stress imaging be accomplished in under 60 s [26]. However, as CMR can detect ischaemia by assessing myocardial perfusion in addition to assessing wall motion abnormalities, treadmill Ex-CMR may be less time sensitive post exercise cessation than treadmill echocardiography. Varying transfer times have been achieved in treadmill Ex-CMR studies (Table 2) [27, 31, 33, 34, 42–45]. Since progression to a CMR compatible treadmill adjacent to the scanner, all studies demonstrate scan initiation in under 30 s, as shown in Video 1 (with the exception of La Fountain et al. in which removal of the face mask assessing oxygen uptake prolonged transfer time [44]) and cine imaging completion in under 60 s [42, 43, 45].

**Table 2:** The transfer times and resultant imaging heart rates in treadmill exercise CMR studies

Study	Year	Patient population	*N*=	Age	Treadmill location	Time (s) from exercise cessation to stress CMR:			Peak HR	Image acquisition HR	CMR completion HR
						Initiation	Cine image completion	Perfusion completion			
Rerkpattanapipat [33]	2003	Patients referred for angiography	27	62 ± 11	Outside MR scanner	NS	61 ± 24	N/A	130 ± 20 bpm	113 ± 16 bpm	NS
Jekic [34]	2008	Healthy volunteers	20	39 ± 15	Corner of MR scanner room	30 ± 4	45 ± 4	57 ± 5^a^	98 ± 7% THR	84 ± 11% THR	NS
Raman [31]	2010	Patients referred for MPS-SPECT	43	54 ± 12	Corner of MR scanner room	42 ± 5	68 ± 14	88 ± 8	93 ± 9% THR	74 ± 10% THR (cine)	NS
Foster [27]	2012	Healthy volunteers	10	23–67	MR Scanner adjacent	24 ± 4	40 ± 7	50.5 ± 9	98 ± 8% THR	86 ± 9% THR	81 ± 9% THR
Thavendiranathan [42]	2014	Healthy volunteers	28	28 ± 11	MR Scanner adjacent	21 ± 2	41 ± 4	N/A	173 (146–196) bpm	148 ± 14 bpm	NS
Sukpraphrute [43]	2015	Patients with known or suspected CAD	115	59 ± 13	Outside MR scanner room	NS	<60s^b^	N/A	88 ± 12% THR	NS	NS
Lafountain [44]	2016	Athletes	10	26 ± 5	Scanner adjacent	36 ± 4^c^	NS	N/A	> 95% THR	87% THR	NS
Raman [45]	2016	Patients referred for MPS-SPECT	94	57 ± 11	Scanner adjacent	25 ± 13	46 ± 16	87 ± 36	97 ± 10% THR	83 ± 11% THR (cine images) 76 ± 11% THR (perfusion)	NS

^a^Time to peak perfusion

^b^100% of patients completed diagnostic imaging in <60s, exact times not specified

^c^CPET face mask removal increased transfer time

*Abbreviations*: *BPM* beats per minute, *CAD* Coronary artery disease, *CPET* cardiopulmonary exercise test, *HR* Heart rate, *MR* magnetic resonance, *N/A* Not applicable, *NS* not specified, *N* Number of patients, *MPS-SPECT* Myocardial perfusion scintigraphy Single-photon emission computed tomography, *THR* Target heart rate


**Additional file 1 Video 1.** Transfer from CMR compatible treadmill to CMR scanner. Example of the transfer process from a CMR adjacent treadmill to the CMR scanner for post stress imaging, as performed by the Ohio State University research group [27].

#### Exercise protocol

**Fig. 2:**
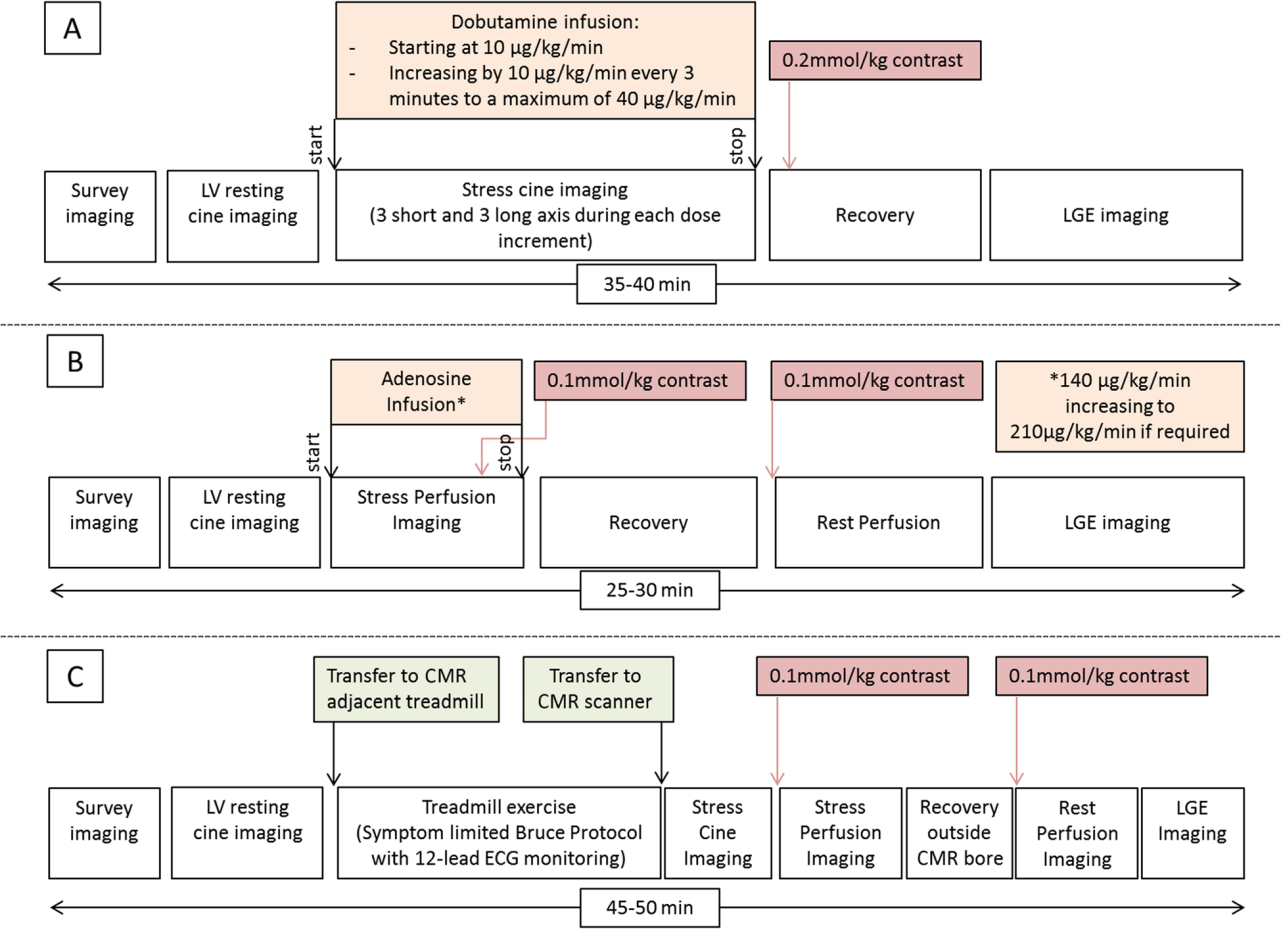
Comparison of pharmacological stress CMR and exercise treadmill CMR protocols. A figure to compare the typical stress protocol for: Dobutamine stress CMR (**a**), adenosine stress perfusion CMR (**b**) and treadmill stress Ex-CMR (**c**). Estimated times of completed protocols may vary and may be dependent on centre experience. LGE = Late gadolinium enhancement; LV = Left ventricle

The current treadmill Ex-CMR protocol entails performing initial resting survey imaging and LV cine imaging. The patient is removed from the scanner bore for a supine 12 lead ECG, then transfers to the scanner adjacent CMR compatible treadmill for an initial standing 12 lead ECG and subsequently performs a symptom limited Bruce protocol treadmill test. After achieving THR > 0.85 x (220-age), the patient is rapidly transferred to the CMR scanner for free-breathing multiplane cine imaging. 0.1 mmol/kg gadolinium contrast is injected prior to stress perfusion imaging, after which the CMR scanner table is removed from the magnet bore to allow 6–8 min of recovery with 12-lead ECG and blood pressure monitoring. The imaging is completed with rest perfusion and late gadolinium enhanced sequences [45]. This protocol is compared with adenosine/dobutamine pharmacological stress CMR imaging in Fig. 2 [20, 45, 46].

CMR imaging sequences used after treadmill exercise have developed, to hasten acquisition and remove breath holding. Initially, retrospectively gated sequences were used with short breath holds to acquire short axis cine imaging for regional wall motion abnormality assessment [33]. The use of real time balanced steady state free precession (bSSFP) imaging with either TSENSE or GRAPPA acceleration, allowed progression to free breathing acquisition of short and long axis LV cine images for regional wall motion assessment [31, 34, 42, 44, 45]. Additionally, perfusion imaging has been performed in several studies, after cine image acquisition, using saturation recovery hybrid gradient echo, echo planar imaging [31, 45].

Treadmill stress CMR offers several benefits over other Ex-CMR modalities (Table 1). Patients often tolerate treadmill exercise greater than cycling, as it is a more natural form of exercise [47] and some patients are unable to cycle [26]. Patients more readily achieve > 85% age predicted THR on the treadmill compared to non-weight bearing exercise [41, 48]. The treadmill test itself provides diagnostic and prognostic information independent of imaging [31, 49–51] and a traditional maximal oxygen uptake assessment is feasible during treadmill exercise within the CMR scanner room [44]. Treadmill stress incorporates a 12-lead ECG exercise test, which is diagnostic even on a CMR adjacent treadmill, compared with non-diagnostic ECG monitoring performed when exercising inside the CMR scanner [52]. This monitoring may be vital to assess for ST segment changes or arrhythmias which can be absolute indications to terminate an exercise test during ischaemia testing [53]. Therefore treadmill Ex-CMR is arguably the safest Ex-CMR methodology to assess CAD. There are limitations to treadmill Ex-CMR. Imaging at numerous exercise intensities is logistically difficult and post stress imaging restricts the time available before a decline in heart rate, thus limiting applications to those achievable within a few minutes. The transfer process also interrupts the advised post-exercise ECG observation period [53]. However, whilst CMR stress perfusion is feasible using the supine cycle ergometer in healthy subjects [54], treadmill Ex-CMR is currently the only Ex-CMR modality to demonstrate utility in ischaemia detection in CAD patients, with clinical evidence from single and multi-centre studies [31, 33, 43, 45].

### In-scanner exercise CMR

In-scanner Ex-CMR can be performed by supine cycle or stepper ergometer, upright cycling in an open magnet, exercise in the prone position or using isometric handgrip; each method is reviewed in Table 1. In-scanner exercise overcomes the main limitation of treadmill Ex-CMR of heart rate reductions between exercise cessation and image acquisition. Imaging during exercise does however have difficulties. Exercise invariably creates movement, increased respiratory motion and interference to the surface ECG, all of which increase with increasing workload. Movement can be reduced with the use of straps or harnesses, but not entirely, especially at higher levels of exercise. Breath held images can be performed during exercise but are non-physiological and difficult at higher exercise intensities [29]. Imaging during free breathing can cause significant though plane motion, making obtaining reliable flow measurements difficult, with the pulmonary trunk especially challenging due to its short length before bifurcation [55]. ECG interference during exercise can create ghosting artefacts with gated images and as previously described, accurate 12 lead ECG monitoring with ST segment analysis cannot be performed during in-scanner exercise [29, 30, 52]. Finally, reaching maximal heart rate is more difficult with supine exercise compared with upright exercise; this is well documented in stress echocardiography with comparisons between treadmill and supine cycle exercise [41, 48, 56, 57]. One explanation is early termination due to leg fatigue [41, 53], thus VO_2max_ is often 10–20% lower in supine cycle exercise than treadmill exercise. Despite this, evidence from stress echocardiography demonstrates equivalency or superiority in detecting ischaemia over post stress treadmill exercise [41, 48, 56]. Indeed, comparatively higher blood pressure attained during supine ergometry [41, 48, 57, 58], results in a similar rate-pressure product to treadmill exercise, such that THR during supine exercise are generally lower when compared to the same exercise intensity in the upright position. Despite the described difficulties of performing in-scanner exercise CMR, techniques have been adapted, with the use of the supine cycle ergometer, such that it is possible to perform in-scanner Ex-CMR to maximal intensity heart rates with imaging during exercise to assess either bi-ventricular function or great vessel flow [29, 55], but often not both, due to time constraints of scanning at incremental levels during or immediately post exercise.

### Supine ergometer exercise CMR

**Fig. 3:**
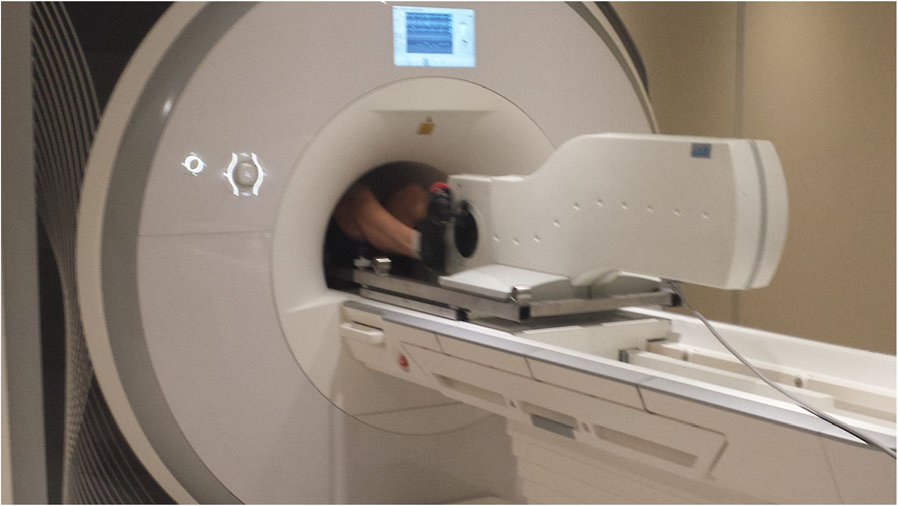
Lode BV supine cycle ergometer during in-scanner supine exercise cardiac magnetic resonance. The Lode BV supine cycle ergometer allows in-scanner exercise, up to maximal exercise intensity, during CMR scanning, as demonstrated by La Gerche et al. [29]. The ergometer attaches firmly to the CMR scanner bed by screw attachments and is safe to use in CMR scanners up to 3 T. The patients’ feet attach into the stirrups and strap securely in place. Resistance can be altered manually in 1-watt intervals. This ergometer is the most utilised modality in Ex-CMR research studies

**Fig. 4:**
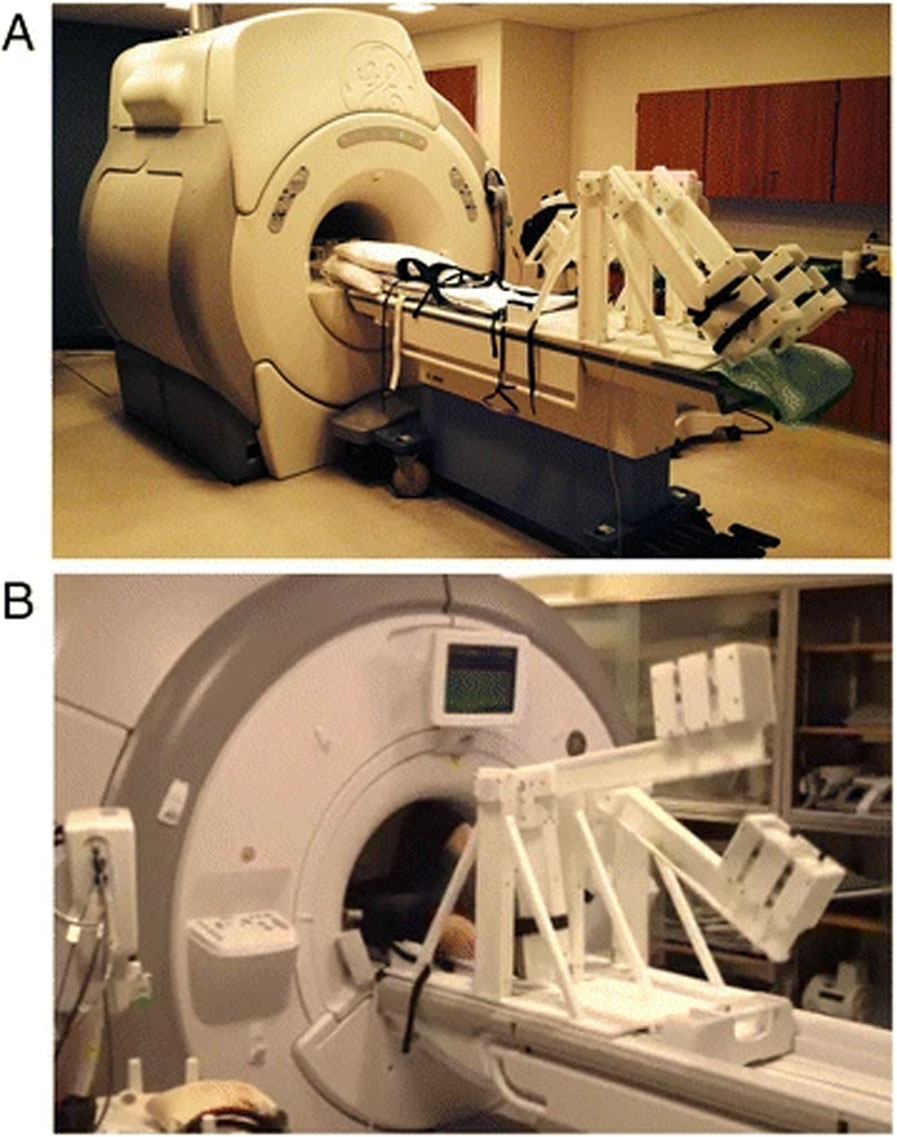
Custom supine stepper ergometer. An example of a supine stepper ergometer utilised in research at the University of Wisconsin [87]. **a**. The ergometer outside the CMR-scanner. **b**- The ergometer in use. The ergometer allows for exercise via an up/down motion, a technique which is reported to cause less movement artefact than the cycle ergometer at the cost of less muscle mass recruitment and thus lower achievable maximal heart rates

The first published use of a CMR compatible cycle ergometer was in 1995 using a 0.5 T whole body scanner to measure exercise changes in aortic flow [59]. Studies utilising commercially produced cycle ergometers followed in 1998 with the use of the Lode BV MR compatible ergometer (Fig. 3) on a 1.5 T CMR scanner [60]. Whilst the majority of Ex-CMR cycle ergometer studies use this system [29, 54, 55, 60–80], some institutions have created custom made CMR compatible cycle ergometers [25, 81, 82]. Other approaches include the supine CMR compatible ‘stepper’ ergometer, that utilises an up/down motion, such as the Lode BV up/down ergometer [83–85], Ergospect cardio-stepper [86] and custom built supine steppers as demonstrated in Fig. 4 [87]. Studies using stepper systems report the benefit of reduced upper body motion, thus reduced motion artefact, and less restriction of leg movement compared with cycle ergometers. However, the up/down motion recruits less muscle mass than the cyclical motion. Thus no study has demonstrated supine ‘stepper’ ergometer Ex-CMR to maximal intensity, as has been demonstrated with supine cycle ergometers (Video 2) [29].

#### Exercise protocol

**Fig. 5:**
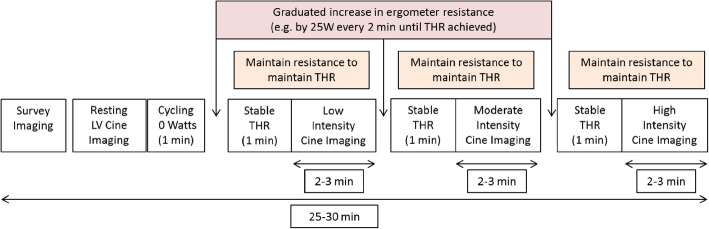
Example of a supine bicycle Ex-CMR protocol. In-scanner Ex-CMR protocols may vary depending on indication, number of exercise stages required and participant fitness. Participants with superior cardiovascular fitness may benefit from shorter intervals between, or more aggressive, increases in resistance to achieve the target heart rate (THR) before leg fatigue. Using the Lode BV supine cycle ergometer, small alterations in resistance are possible, which can assist a tight control of THR

Exercise protocols used with supine cycle ergometer Ex-CMR vary depending upon the aims of the study/investigation. An example protocol is presented in Fig. 5. The number of stages of exercise is variable depending upon the aims. A typical protocol often entails a period of supine cycling with no resistance (0 W) on the ergometer, to allow the patient to accustom to the cycling positon and the advised cadence. This is followed by a graduated increase in resistance, for example by 25 W every 2 min, until THR is achieved. However, in athletes a faster increase in resistance may be advised. The resistance is then maintained whilst at the specific exercise intensity heart rate. Indeed minute changes in resistance can be made, if required, to ensure tight control of heart rate during scanning. Once THR has been maintained for 60 s CMR imaging will commence. After completion of a specified exercise intensity stage, the process of ‘ramping up’ resistance, acquiring a stable THR prior to imaging and maintaining that THR during imaging is repeated for each exercise stage required.

#### Ventricular volumes

Cycle ergometer Ex-CMR ventricular volume assessment has progressed from imaging during exercise cessation with breath holding [25, 67, 70–72, 81], breath holding during exercise [82, 86], free breathing with exercise cessation [54, 64, 66], to free breathing during continuous exercise [29, 83]. Initial studies utilised turbo field echo planar imaging (EPI) with retrospective gating to acquire short axis cine imaging for biventricular volumes during exercise [67, 70, 71]. Subsequent studies using retrospective ECG gating have used balanced steady state free precision (bSSFP) sequences [25, 81, 82, 88, 89]. Recently, an Ex-CMR study used a 3 T scanner to assess LV volumes similarly used bSSFP sequences to acquire 4 chamber and 2-chamber cines to calculate LV function via Simpson’s bi-plane method [86].

To date, published retrospective gating techniques have not performed image acquisition with free breathing during exercise; this has been achieved with real time techniques. Lurz et al. first demonstrated the feasibility of imaging with free breathing during continuous exercise, assessing biventricular volume and function in healthy subjects. A radial *k-t* SENSE real-time sequence demonstrated higher temporal resolution short axis cine images than a standard vendor supplied real time sequence in patients exercised to submaximal intensity [83]. Recently, a re-binning real time sequence with automated motion correction showed similar LV volumes, but improved signal-to-noise ratio (SNR) and temporal resolution than conventional real time technique during Ex-CMR to moderate intensity [90]. Real time imaging enables free breathing during Ex-CMR, however as the ECG is required to retrospectively reconstruct the images, it still suffers from gating issues at maximal exercise intensities. Indeed patients in the re-binning study were excluded as the images could not be reconstructed due to ECG interference [90]. Le et al. preferred exercise cessation with a real time sequence, to overcome ECG interference, to assess patients exercised to fatigue [66].

**Fig. 6:**
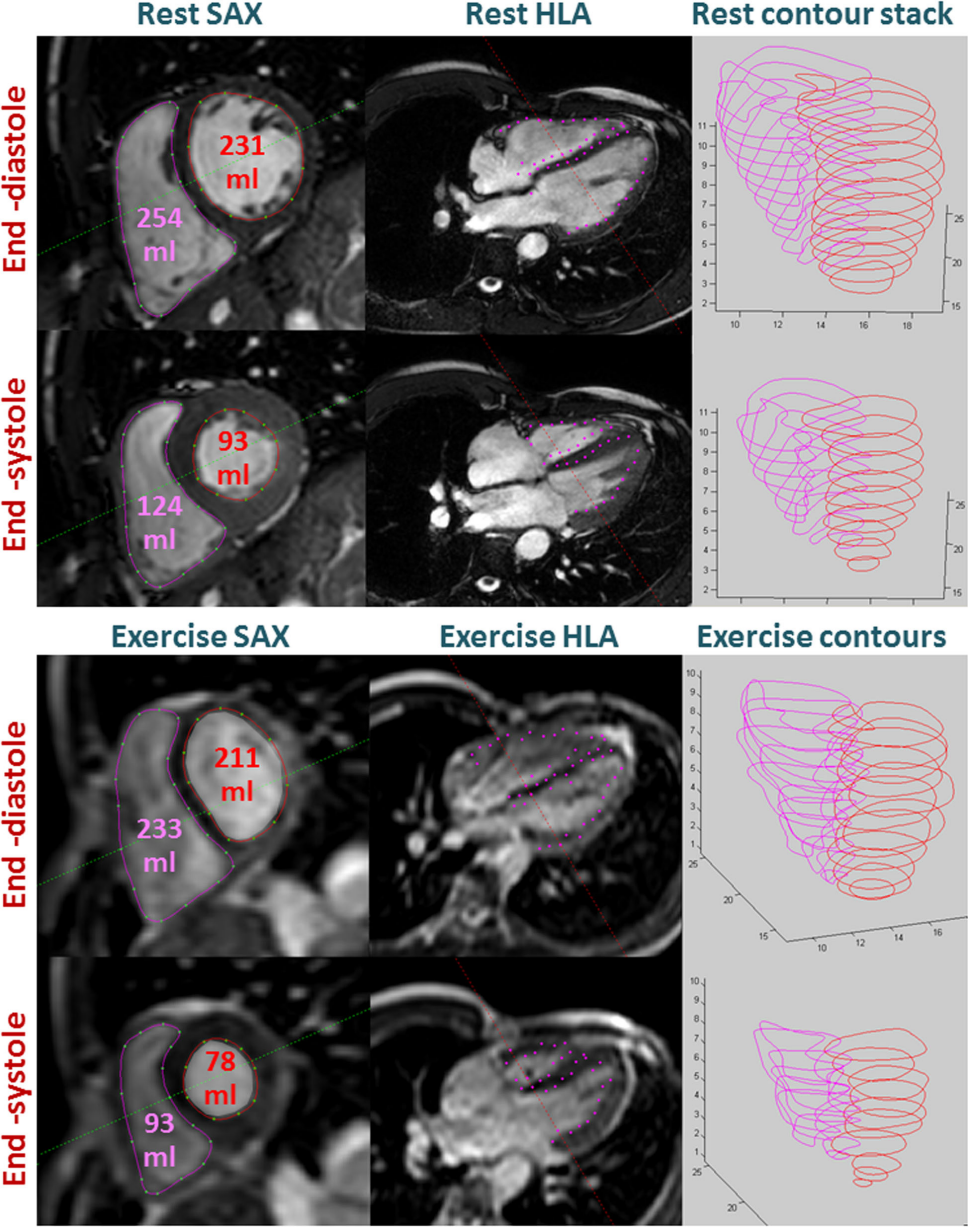
Example of real-time ungated CMR imaging at rest and during maximal exercise. Real-time ungated biventricular volume assessment methodology as developed by La Gerche et al. and subsequently utilised in numerous subsequent clinical studies

Reliable biventricular assessment during maximal exercise has been achieved by La Gerche et al. with the development of an ungated real time sequence (Fig. 6). Video 2 demonstrates in-scanner exercise and resultant cine imaging by this technique. The technique utilises specialized in-house software to compensate for respiratory motion which overcame the issue of excessive ECG artefact encountered during high intensity exercise with the comparator gated sequence. The cardiac output derived by this technique was validated against the direct Fick method with excellent agreement [29]. Although a limitation of this approach is prolonged post processing time and the requirement for bespoke in-house analysis software to synchronize the ECG and respiratory movement with the images, it is the only method to date to allow accurate biventricular quantification during maximal exercise and has since been utilised in a number of clinical studies [62, 63, 65, 73, 76, 77, 80].

It should be noted that the physiological response to exercise can differ depending on exercise type (aerobic/anaerobic/dynamic) and position (upright/semi-supine/supine) [53]. Previous non-CMR exercise studies have published contradicting LV end diastolic volume (EDV) responses to supine exercise, demonstrating an increase [91], a decrease [92] or no significant change [93–95] in EDV with exercise. However, a recent meta-analysis of LV function during supine Ex-CMR, involving a pooled analysis of 16 studies, demonstrated a significant rise in LV ejection fraction (EF) with exercise, driven by a fall in end systolic volume (ESV), whilst EDV remained unchanged [86].

#### Flow acquisition

Ex-CMR studies for flow assessment began by imaging during cessation of exercise and have progressed to free breathing acquisition during continuous exercise. Ex-CMR studies have predominantly assessed aortic and/or pulmonary artery flow, however flow assessment of the superior vena cava (SVC), left and right pulmonary arteries and all four pulmonary veins are feasible [96]. Inferior vena cava (IVC) flow assessment via Ex-CMR is challenging, owing to significant diaphragmatic movement during exercise, however has been achieved using real time phase contrast imaging with a novel respiratory compensation protocol that allows accurate assessment of the respiratory cycle via assessing chest wall motion [97]. Ex-CMR flow assessments were first performed in 1995 at 0.5 T using spiral EPI with breath holds after cessation of exercise to demonstrate increases in descending thoracic aorta flow, images were low resolution and heart rates equivalent of low exercise intensity [59]. Faster imaging techniques were adopted to reduce breath-hold times, allowing flow imaging after cessation of higher intensities of exercise, initially with the use of EPI in healthy subjects [68] and subsequently patients with congenital heart disease [67, 98].

Ex-CMR flow acquisition with free breathing during exercise was first performed by Niezen et al. in 1998 using retrospectively gated phase contrast velocity encoding during low intensity exercise [60], and subsequently during moderate exercise intensity [69, 74] and post exercise cessation [96]. Retrospective gating via pulse oximetry is commonly used to overcome ECG gating artefact at higher exercise intensities [60, 69].

Real time flow imaging techniques have been used successfully in Ex-CMR with the aid of post hoc analysis, with either in-house plug-ins for open source software or in-house developed specialist software. Steeden et al. used a spiral phase contrast real time sequence accelerated with sensitivity encoding (SENSE) to acquire aortic flow and a radial KT SENSE sequence to assess LV volumes, during light/moderate exercise on the Lode BV (Up/Down) ergometer. Aortic flows acquired by the real time technique had good agreement at rest with a standard 2D retrospective free breathing flow acquisition technique and at rest and during exercise with the stroke volume from LV volumes [99]. The same research group then utilised real-time unaliasing by Fourier-encoding the overlaps using the temporal dimension and sensitivity encoding spiral phase-contrast magnetic resonance sequence (UNFOLDed-SENSE) in subsequent studies. Initially the UNFOLDed-SENSE aortic flow sequence was used in a CMR augmented cardiopulmonary exercise test (CMR-CPET) to demonstrated feasibility of CMR-CPET in healthy adult subjects [84] and subsequently in combination with real time k-t SENSE short axis cines to perform CMR-CPET in paediatric healthy controls, repaired tetralogy of Fallot (ToF) and pulmonary artery hypertension patients [85]. A limitation of this continuous flow technique, highlighted by the authors, is the need to continuously measure flow to guarantee acquisition of data at peak exercise. This results in acquiring ≤25,000 frames of flow images and therefore creates a significant reconstruction and post processing problem. As such, the reconstruction requires an online graphics processing unit reconstruction system and in-house post processing tool to cope with the volume of data [85]. Studies by Aschenfeldt and Heiberg et al. similarly used real time flow acquisition but with fast EPI and a half scan factor of 0.6, with analysis on in-house developed specialist software. The technique allowed aortic and pulmonary flow acquisition at numerous exercise levels and to ‘true’ submaximal intensity (> 85% age predicted maximum heart rate ), having being used to assess healthy subjects and patients with surgically repaired ventricular septal defects (VSD) [55, 61]. Recently, ungated real time biventricular volume and aortic and pulmonary flows were performed during exercise to moderate exercise intensity in healthy subjects and patients with pulmonary arterial hypertension, the flow volumes acquired were similar to stroke volumes acquired from biventricular volumes [100].

### Upright cycle ergometer

Cheng et al. demonstrated the feasibility of assessing pulmonary artery flow during continuous exercise to moderate intensity in adults and children in a 0.5 T vertical open bore scanner [101]. Although upright cycling may be more tolerated than supine exercise, it requires the use of an open low field CMR scanner, with benefits of easing claustrophobia, but inherent issues of lower SNR. CMR is feasible at lower field strengths [102], however, although the scanners are commercially available they are not in mainstream use, as such very few Ex-CMR studies have utilised this modality.

### Isometric handgrip stress CMR

Isometric exercise involves the contraction of skeletal muscle without the elongation of the muscle, as such is also called static exercise [103]. This is feasible during CMR by IHG or isometric bicep exercise [104]. IHG exercise comprises the constant squeezing of a lever on a hand dynamometer, generally to a percentage of the subjects maximum force. The technique only allows for modest increases in heart rate, typically 10-20 bpm above resting rates, but causes minimal movement. As such the technique has mainly been used for CMR spectroscopy (MRS) or coronary artery flow imaging where minimal movement artefact is pivotal and minimal heart rate increases are acceptable. Weiss et al. performed the seminal work with IHG-Ex CMR, developing the ^31^P CMR spectroscopy (CMRS) stress test which remarkably can detect ischaemia in patients with CAD, despite minimal stress and increases in heart rate [105].

### Prone exercise CMR

Prone Ex-CMR was first employed by Conway et al. who performed Ex-CMR studies using knee extension with a custom system of straps, cables, pulleys and weights [106]. Numerous subsequent Ex-CMR studies have similarly used prone Ex-CMR performing alternative knee flexion with ankle weights [107–109]. Low-moderate intensity exercise is feasible by this approach, with the most significant response from the custom knee extension system by Conway et al., with a mean stress heart rate of 119 bpm. This technique has other inherent limitations; exercising whilst prone is an unnatural form of exercise which uses weights or resistance bands attached to the legs, increasing the resistance can be labour intensive, requiring alterations during exercise/scanning or exercise cessation. Prone Ex-CMR often requires an auditory cue from a metronome to determine work speed, however if this isn’t strictly adhered to, then the exact workload is unknown. As such, only Conway et al. employed incremental resistance by increasing the attached weights to the pulley system used. As such, prone Ex-CMR is not ideal to assess incremental levels of exercise intensity or where strict heart rate increases or levels are required.

## Exercise CMR in specific disease conditions

Ex-CMR has been utilised to study a wide range of cardiovascular pathology from CAD to potential cardiomyopathic conditions and structural/congenital heart disease. The following provides an overview of the more common disease areas investigated.

### Coronary artery disease

Numerous studies have assessed regional wall motion and/or myocardial perfusion with Ex-CMR, all utilising treadmill exercise with post-stress imaging to achieve this (Table 3) [27, 31, 33, 34, 42, 44, 45]. Supine cycle ergometer myocardial stress perfusion has been demonstrated as feasible in healthy subjects [54] but has not yet been utilised to assess patients with CAD. The original treadmill work assessed 27 patients referred for coronary angiography with treadmill stress CMR and demonstrated a sensitivity and specificity of 79 and 85% respectively, for detecting stenosis (> 70%) [33]. The Ohio State University research group developed a CMR compatible treadmill [34] and utilised this in the EXACT trial [45]. The EXACT trial (exercise CMR’s accuracy for cardiovascular stress testing) is a multicentre prospective observational study. Four participating centres recruited 210 patients who were clinically referred for treadmill radionuclide single photon emission computed tomography (SPECT) and directly compared this with treadmill stress CMR. Patients had a rest ^99m^Tc SPECT rest scan followed by resting CMR cine images. An in room treadmill stress test was then performed with ^99m^Tc tracer injected at peak stress. Patients were then rapidly transferred to the CMR scanner for CMR stress images, including cine and myocardial perfusion imaging. Stress SPECT images were subsequently performed in an adjacent gamma camera. All images were acquired in the same day from the same treadmill stress. Ninety-four of the patients had coronary angiography (X-ray or CT coronary angiography). This multi-centre trial demonstrated superiority of Ex-CMR over exercise SPECT for identifying > 70% at coronary angiography. Sensitivity, specificity, positive and negative predictive values for Ex-CMR were 78.6, 98.7, 91.7 and 96.3% respectively, compared to 50, 93.7, 58.3 and 91.5% respectively for exercise SPECT. The EXACT trial was the first, and to date the only, multicentre Ex-CMR trial and demonstrated the diagnostic capabilities and clinical utility of treadmill Ex-CMR in CAD. The initial and downstream costs of treadmill Ex-CMR have being assessed by the recently completed and under review, multicentre EXACT-COST trial, which randomised patients to either treadmill stress SPECT or treadmill Ex-CMR. Treadmill Ex-CMR has similarly shown a promising comparison with exercise echocardiography in a pilot study of 28 patients, with Ex-CMR showing more myocardial segments being adequately visualised than by exercise echo [42]. In addition to CAD diagnosis, treadmill stress Ex-CMR has potential prognostic utility in predicting adverse events in those with known or suspected CAD. In a study of 115 patients with an indication for stress imaging, wall motion abnormalities were assessed at rest and post treadmill stress via Ex-CMR. The presence of stress induced regional wall motion abnormalities on treadmill Ex-CMR better identified those at risk of future events (death, myocardial infarction & unstable angina prompting admission) when compared to exercise ECG alone [43].

**Table 3:** Features and findings of treadmill exercise Cardiac MRI studies in coronary artery disease

Study	Patient population	n.	Mean Age (years)	Treadmill location	Findings
Rerkpattanapipat (2003) [33]	Patients referred for coronary angiography	27	62 ± 11	Outside scanner room	Detected coronary artery stenosis > 70% on coronary angiography with sensitivity and specificity of 79% & 85%.
Raman (2010) [31]	Patients referred for SPECT	43	54 ± 12	MR scanner room corner	Exercise stress CMR is feasible with cine wall motion and perfusion assessment and has moderate agreement with SPECT (K = 0.58)
Sukpraphrute (2015) [43]	Patients with known or suspected CAD	115	59 ± 13	Outside scanner room	Treadmill Ex-CMR RWMA assessment identified those at risk of future adverse events (myocardial infarction, death, unstable angina prompting admission) 47% with RWMA vs 17% without
Raman (EXACT trial) (2016) [45]	Patients referred for SPECT	94	59 ± 13	Scanner adjacent	Treadmill stress CMR demonstrated a stronger correlation with coronary angiography and superior specificity, sensitivity, positive and negative predictive values for > 70% stenosis at angiography than treadmill SPECT

*Abbreviations*: *CAD* coronary artery disease, *CMR* cardiovascular magnetic resonance, *RWMA* regional wall motion abnormality, *SPECT* single photon emission computed tomography,

A recent supine cycle Ex-CMR feasibility study demonstrated post exercise T1 mapping could be a surrogate marker in detecting myocardial blood flow. In 14 CAD patients, post exercise T1 reactivity was associated with the severity of myocardial perfusion defect on MPS-SPECT. Therefore, Ex-CMR T1 mapping shows potential for detecting myocardial ischaemia [110].

Ex-CMR studies using IHG stress can reproducibly assess coronary artery endothelial function by assessing coronary artery cross-sectional area change and coronary flow by velocity encoded CMR [111]. Studies have demonstrated endothelial dysfunction in CAD in the form of paradoxical vasoconstriction with exercise [112], assessed the reproducibility of this technique [113] and shown that the normal vasodilatory response of healthy coronary arteries is mediated by nitric oxide [114]. Coronary artery blood flow assessment after supine bicycle exercise is also feasible, correlating well with invasive measures [115], and has been used to demonstrate, in a randomised double-blinded crossover trial, that hormone replacement therapy and high dose statins increase coronary artery blood flow in postmenopausal patients without CAD [116]. Thus, Ex-CMR may have a future role in research studies investigating CAD pathogenesis in low risk populations.

### Athletic heart disease

Ex-CMR studies show promise in providing an additional tool to differentiate the athletic heart adaptation from cardiomyopathy, and for risk stratifying endurance athletes against RV arrhythmias. Ex-CMR to maximal intensity on 14 endurance athletes after a 150 km cycle race, showed that the acute RV dilatation and reduced RV ejection fraction (RVEF), previously demonstrated with endurance exercise, worsened during further exercise and was not the result of confounding post-race variables such as increased afterload and autonomic activation [75]. Using Ex-CMR, endurance athletes with RV arrhythmias demonstrated more RV dysfunction during exercise, despite normal resting RV function, than athletes without RV arrhythmias and healthy controls [65]. In terms of differentiating the athletic heart from cardiomyopathy, when 10 healthy endurance athletes were compared with 9 endurance athletes with subepicardial fibrosis and 5 patients with dilated cardiomyopathy, they demonstrated a significantly greater augmentation of LV ejection fraction (LVEF) during exercise of 14 ± 3%, compared with 4 ± 3% and 5 ± 6% respectively. The endurance athletes with subepicardial fibrosis had similar resting haemodynamics and exercise capacity to the healthy endurance athletes, suggesting that Ex-CMR may be helpful in differentiating healthy endurance athletes from those with fibrosis [63].

### Congenital heart disease

CMR has significant benefits over alternative non-invasive imaging modalities for the assessment of congenital heart disease, especially for complex lesions and right heart pathologies. These benefits have been utilised with supine ergometer Ex-CMR to assess the physiological responses to exercise in a variety of conditions (Table 4).

**Table 4:** Supine ergometer exercise CMR studies in Congenital Heart Disease

Study	n.	Population	Variable assessed	Exercise intensity*	Imaging Technique	Findings
Pedersen (2002) [98]	11	Children with prior TCPC operation	SVC, IVC tunnel, LPA & RPA flow	Low-Moderate	TFEPI Retrospective gating Breath hold Exercise cessation	IVC flow doubled with exercise with equal distribution to both lungs, suggesting pulmonary resistance rather than geometry decides exercise flow distribution in the TCPC circulation
Roest (2002) [117]	31	Repaired ToF (15) & healthy volunteers (16)	Biventricular volume and pulmonary flow	Moderate		Repaired ToF patients demonstrated a decrease in PR with exercise but abnormal RV response to exercise compared to healthy controls.
Roest (2004) [71]	41	Atrial corrected-TGA (27), Healthy control (14)	Biventricular volumes	Moderate		Patients with atrial correction of TGA demonstrate abnormal biventricular response to exercise despite normal resting function.
Oosterhof (2005) [67]	64	Atrial corrected TGA (39) & Healthy volunteers (25)	Aortic flow and systemic ventricle function (exercise vs dobutamine stress)	Vigorous		A trial corrected TGA patients demonstrate an abnormal response to exercise with a decrease in systemic ventricle EF, but a normal response with dobutamine stress. Therefore these two methods cannot be used interchangeably in this group.
Lurz (2012) [118]	17	PPVI for PR/PS as a result of congenital heart disease	Biventricular volumes	Until exhaustion pre-PPVI**	Realtime radial K-T sense volumes	Post PPVI, PS patients had restoration of RVEF exercise reserves, PR patients only had a mild augmentation of exercise SV.
Van De Bruaene (2014) [78]	10	Fontan circulation (10)	Systemic ventricle volumes, invasive radial and PA pressures	Submaximal	Un-gated real time, free-breathing.	Sildenafil improves cardiac index during exercise in Fontan patients suggesting pulmonary vasculature resistance is a physiological limitation in this patient group.
Van De Bruaene (2015) [79]	10	Fontan circulation (10)	Systemic ventricle volumes, invasive radial and PA pressures	Submaximal		Demonstrated that systemic ventricular filling increases with inspiration, ‘respiratory pump’, which persisted throughout exercise.
Khiabani (2015) [119]	30	Fontan circulation	Ascending and descending aortic flow and SVC flow	Moderate/ to VAT	Retrospective gating, breath hold after exercise cessation	Computational fluid dynamics simulations performed on the measured flows demonstrated that power loss in the TCPC circulation increased exponentially as patients exercised towards ventillatory anaerobic threshold (VAT)
Barber (2016) [85]	30	Pediatric: Repaired ToF (10) i-PAH (10) Control (10)	MR-CPEX Biventricular volumes & aortic cardiac output	Submaximal	Realtime radial K-T sense volumes Realtime UNFOLDed-SENSE flow	MR-augmented CPEX is feasible and safe in children with cardiac disease. Peak VO2 was reduced in children with PAH or repaired ToF compared with healthy controls.
Wei (2016) [97]	11	Fontan circulation/TCPC	IVC, SVC and aortic flows	Moderate/ to VAT	Realtime shared velocity encoded EPI	Utilised a novel chest wall tracking technique to demonstrate respiration caused minimal net changes in mean flow, thus validating the routine use of breath held imaging in these patients and that IVC and descending aortic flows were interchangeable.
Asschenfeldt (2017) [61]	40	Surgically repaired VSD (20) and control (20)	Aortic and pulmonary flow	Submaximal	Real time EPI with half-scan FB during exercise	Patients demonstrated impaired cardiac index vs controls related to increased retrograde flow in pulmonary artery with progressive exercise.
Tang (2017) [120]	47	Fontan circulation/TCPC	SVC, ascending and descending aortic flows	Moderate/ to VAT	Free breathing Exercise cessation	Fontan patients with a smaller TCPC diameter index (which accounts for narrowing’s in the TCPC circulation) demonstrate increased indexed power loss and worse exercise performance.
Habert (2018) [121]	22	Repaired ToF (11) Control (11)	Biventricular volumes & aortic distensibility	Low-moderate	Breath hold exercise cease	Repaired ToF demonstrated reduced bi-ventricular contractile reserve and reduced ascending aortic distensibility vs controls.
Helsen (2018) [80]	45	Atrial corrected-TGA (23) CC-TGA (10) Control (12)	Systemic ventricle volumes	Maximal	Un-gated real time, free-breathing.	A trial corrected-TGA patients demonstrate deteriorating systemic ventricle volumes and stroke volume during exercise compared with CC-TGA patients; caution should be used in analysing pooled systemic right ventricle populations.
Jaijee (2018) [100]	48	PAH (14) Control (34)	Biventricular volumes. Aortic and pulmonary flow	Submaximal		PAH patients demonstrated a decrease in RV contractile reserve with exercise and healthy controls had a reduced contractile reserve exercising during hypoxia (breathing 12% oxygen)
Claessen (2019) [77]	30	Fontan (10), Control (20)	Systemic ventricle volumes, invasive radial and PA pressures	Maximal		Fontan patients have a diminished heart rate reserve as a result of abnormal cardiac filling rather than sinus atrial node dysfunction causing chronotropic incompetence.

*Exercise intensities according to American college of sports medicine guidelines

**Patients exercised until exhaustion pre-PPVI, then post-PPVI patients exercised to the same exercise intensity as pre-PPVI

*Abbreviations*: *CC* congenitally corrected, *BH* breath hold, *FB* free breathing, *i-PAH* idiopathic pulmonary hypertension, *IVC* inferior vena cava, *LPA* left pulmonary artery, *RPA* right pulmonary artery, *SVC* superior vena cava, *TGA* transposition of the great arteries, *ToF* tetralogy of Fallot, *VAT* ventillatory anaerobic threshold, *VSD* ventricular septal defect

Despite normal resting biventricular function, patients with prior atrial correction for transposition of the great arteries (TGA) demonstrated little or no increase in EF & stroke volume (SV) on Ex-CMR compared with healthy controls. The abnormal RV response to exercise correlated with exercise intolerance in these patients [71]. A subsequent study comparing haemodynamic response between Ex-CMR and dobutamine stress CMR also demonstrated no increase in SV and EF with exercise in atrial-corrected TGA patients, but a significant increase of SV and EF in controls during exercise and both groups during dobutamine stress CMR. This study demonstrated the two stress techniques could not be used interchangeably in this cohort of patients and re-affirmed the abnormal exercise response in atrial-corrected TGA patients. The differences in stress response between dobutamine and exercise are likely due to the differing effects on pre and afterload and could be present in other cardiac disease processes. Therefore, the type of stressor must be taken into account when interpreting the different methods [67]. A subsequent Ex-CMR study demonstrated a divergent cardiac response during Ex-CMR between atrial corrected TGA and congenitally corrected TGA (CC-TGA) patients, demonstrating the need for caution in performing pooled analysis of systemic RV (s-RV) patients. At rest, atrial corrected TGA patients had significantly higher global circumferential strain but similar global longitudinal strain and s-RVEDVi and ejection fraction. CC-TGA patients demonstrated more pronounced septal interstitial expansion. During Ex-CMR, atrial corrected TGA patients demonstrated deteriorating s-RVEDVi and RV SV during exercise compared with an unchanged s-RVEDVi and augmented RV SV in CC-TGA patients [80].

Ex-CMR has been important in demonstrating causes of exercise intolerance in patients with Fontan circulation. Pedersen et al. investigated patients with total cavopulmonary connection (TCPC), a palliative Fontan type operation, using supine bicycle Ex-CMR. At rest blood flow was lower to the smaller left lung. During exercise the flow increased proportionally with systemic venous return, IVC flow doubled and SVC flow increased slightly. The ratio of flow distribution to the lungs was unchanged with exercise, contrary to earlier resting studies demonstrating preferential streaming of IVC flow into the left pulmonary artery (LPA) and SVC to the right pulmonary artery (RPA) [122, 123]. The study concluded that pulmonary vascular resistance, rather than anastomosies geometry, was the major factor effecting exercise flow distribution and thus exercises intolerance [98]. This assertion was further demonstrated in an Ex-CMR study in 10 Fontan patients, in which rest and exercise systemic volumes were assessed with simultaneous invasive radial artery and pulmonary artery pressure measurements before and after sildenafil. Sildenafil improved cardiac index during exercise with a decrease in total pulmonary resistance index and an increase in stroke volume index [78]. The ‘respiratory pump’ theorized in the Fontan circulation has been demonstrated in 10 Fontan patients who showed increased ventricular filling during inspiration which persisted during Ex-CMR. This may provide a rationale for respiratory muscle training in this patient group [79]. Subsequently, Wei et al. studied the significance of varying respiratory effects on flow in the TCPC anatomy by using real time phase contrast imaging to assess the SVC, IVC, ascending and descending aortic flow during breath holding, free breathing and exercise conditions. The study demonstrated that whilst respiration affects IVC and SVC waveform, it did not significantly affect mean flow. The study therefore validated using breath held images, which benefit from reduced image artefacts, for routine mean flow assessment in TCPC anatomy patients. Additionally, the study demonstrated interchangeable mean flows between the IVC and descending aorta, justifying the use of descending aorta flow as a surrogate for IVC flow, which is difficult to assess during exercise due to diaphragmatic motion, in TCPC anatomy patients [97]. Khiabani et al. demonstrated the association of exercise performance with power loss in TCPC anatomies when performing an Ex-CMR study in 30 patients with Fontan circulation. Power loss calculations made from computational fluid dynamic simulations were performed on the in vivo TCPC anatomies and acquired flows, demonstrating that as cardiac output increased during exercise, power loss increased exponentially [119]. A subsequent Ex-CMR study demonstrated an inverse correlation between the TCPC diameter index, which accounts for vessel narrowing in the TCPC, and indexed power loss during Ex-CMR to the ventillatory anaerobic threshold, thus suggesting that reducing vessel narrowing and elevated power loss could improve exercise capacity and quality of life in patients with TCPC [120]. The diminished heart rate reserve often seen in Fontan patients was investigated in a recent Ex-CMR study and demonstrated as a likely secondary phenomenon rather than due to sinus atrial node dysfunction. 10 Fontan patients demonstrated an increased chronotropic response to exercise but an early plateau in cardiac output, compared with 20 healthy controls, caused by premature reductions in ventricular filling and stroke volume. This Ex-CMR study thus determined that the diminished heart rate reserve observed in Fontan patients was a result of abnormal cardiac filling rather than sinus atrial node dysfunction causing chronotropic incompetence [77].

Patients with prior surgically repaired VSD commonly have reduced functional capacity and often demonstrate resting retrograde ascending aortic flow. A recent Ex-CMR study demonstrated a decreased cardiac index in young adults exercised to submaximal intensity. This was determined secondary to a previously unrecognised increase in retrograde pulmonary flow with exercise along with previously demonstrated chronotropic incompetence in this patient group [61].

Ex-CMR has been demonstrated as feasible in repaired ToF in both adults [121] and children [85]. The seminal Ex-CMR study in repaired ToF patients by Roest et al. demonstrated an abnormal RV response to exercise compared with controls, but a decrease in pulmonary regurgitation [117]. A more recent study demonstrated reduced biventricular contractile reserve and aortic distensibility in repaired ToF patients compared to healthy subjects during Ex-CMR, which may be an early sign of increased aortic rigidity [121].

An Ex-CMR study assessing the exercise biventricular response to percutaneous pulmonary valve implantation (PPVI) in patients with either pulmonary regurgitation or pulmonary stenosis of heterogenous congenital aetiologies, demonstrated that PPVI resulted in restoration of RVEF exercise reserves in pulmonary stenosis patients but only a mild augmentation of exercise SV post PPVI in pulmonary regurgitation patients [118].

### Pulmonary hypertension

Idiopathic PAH patients have shown worsened RV function and a failure to augment bi-ventricular stroke volumes during Ex-CMR when compared with healthy subjects [64]. A recent study found similar differences between healthy controls and PAH patients, but additionally, when healthy controls were exercised during hypoxia (breathing 12% oxygen), which is associated with transient acute pulmonary hypertension, this resulted in decreased RV contractile reserve, but not as dramatically as PAH patients exercising during normoxia [100]. A further increase in pulmonary artery pressure during exercise in idiopathic PAH patients is the presumed cause of worsened RV contractile reserve; this was demonstrated in an Ex-CMR study of 15 patients with chronic thromboembolic pulmonary hypertension (CTEPH), 7 patients post pulmonary endartectomy (PEA) and 14 healthy controls. Despite a normal resting RVEF and mean pulmonary artery pressure post PEA, the RVEF and pulmonary compliance decreased with exercise in the post-PEA group along the CTEPH group. This was in contrast to healthy subjects whose RVEF increased with exercise and pulmonary compliance only mildly decreased. Treatment with sildenafil in the post-PEA group did not alter resting RVEF or haemodynamics, but significantly decreased exercise mean pulmonary artery pressure and increased exercise RVEF. Thus this Ex-CMR study demonstrated that post-PEA patients display an abnormal pulmonary vascular reserve which can be partially reversed by Sildenafil [76].

### Diabetes

Ex-CMR studies in diabetes mellitus have demonstrated that adolescents with type 1 diabetes mellitus (T1DM) have a reduced exercise capacity, and resting and exercise LV stroke volumes (which correlated to glycaemic control), compared with non-diabetic controls [88]. The same group demonstrated improved, but not normalised, LVEF in T1DM adolescents who undertook regular intense exercise for 20 weeks when compared with non-exercising T1DM controls, suggesting that the diastolic dysfunction could be partially reversed by regular intense exercise [89]. Impaired cardiac function during exercise was similarly demonstrated in adolescent type 2 diabetes mellitus (T2DM) patients when compared with obese non-diabetics and controls [81]. Similar to the studies in T1DM, the T2DM adolescents demonstrated smaller LV SV, EDV and ESV at rest and during exercise, with a smaller increase in EF than non-diabetic controls. As such, all 3 studies demonstrated a decreased cardiac reserve during exercise in adolescent diabetics, whether T1DM or T2DM, when compared with non-diabetic controls [81, 88, 89].

### Valve disease

Ex-CMR studies in aortic regurgitation (AR) have demonstrated that isolated AR in children and adults decreased during ‘steady state submaximal exercise’ CMR, which equated to prolonged light in-scanner exercise on a custom built device [124]. Roberts et al. assessed the short term effects metoprolol and losartan had on exercise haemodynamics in chronic AR patients after supine exercise in a cross-over study, showing that with metoprolol there was a lower heart rate, greater AR regurgitant fraction, lower aortic distensibility and greater indexed LV EDV and ESV compared to Ex-CMR on losartan [125].

## Exercise CMR spectroscopy

Phosphorus CMRS (^31^P-CMRS) is a non-invasive means of assessing the myocardial phosphocreatine to adenosine triphosphate (ATP) concentration ratio (PCr/ATP) and a sensitive indicator of myocardial energy status. Numerous exercise ^31^P-CMRS studies have been performed. As minimal movement is essential, the majority of studies have employed IHG or prone exercise. Although the feasibility of supine cycle ergometer exercise ^31^P-CMRS was demonstrated in healthy subjects in 1994, this technique has not since progressed to assessment of cardiac disease [126].

### Isometric hand grip ^31^P-CMRS

Weiss et al. performed the initial study in exercise CMRS, developing the ^31^P CMRS stress test by performing CMRS before, during and after isometric hand grip exercise in patients with CAD, non-ischaemic heart disease and healthy controls. The study demonstrated a deceased regional PCr/ATP ratio during exercise in patients with > 70% stenosis in the left anterior descending coronary artery (LAD) or left main coronary artery vs no change in healthy controls and patients with non-ischaemic heart disease. The decreased PCr/ATP ratio improved 2 min after cessation of exercise and did not recur in those that had subsequent revascularization [105]. The technique was further validated by Yabe et al. as a sensitive method to detect myocardial ischaemia in those with reversible defects on ^201^T1 MPS-SPECT [127] and subsequently used to demonstrate decreased PCr/ATP ratios on exercise in cardiac transplant patients [128] and patients with Chagas heart disease [129].

### Prone exercise ^31^P-CMRS

Prone exercise ^31^P-CMRS has been used to investigate patients with T2DM and separately patients with hypertrophic cardiomyopathy (HCM). Resting ^31^P-CMRS studies have demonstrated decreased energy metabolism via a decreased PCr/ATP ratio in T2DM patients with functionally normal hearts [130]. A study by Levelt et al., employed prone knee flexion exercise stress to perform ^31^P-CMRS and an adenosine stress CMR to assess myocardial perfusion and oxygenation. The T2DM patients demonstrated a lower resting PCr/ATP ratio which decreased further with exercise compared to controls and a decrease in myocardial perfusion and oxygenation. The correlation between exercise myocardial perfusion and oxygenation and PCr/ATP ratio at exercise suggested that coronary microvascular dysfunction exacerbated the cardiac energetics during exercise [109].

Dass et al. used prone knee flexion exercise stress to perform ^31^P-CMRS and an adenosine stress CMR to assess myocardial perfusion and peak filling rates in patients with HCM and healthy controls. The HCM patients demonstrated a lower resting PCr/ATP ratio which decreased further with exercise compared to controls, a decrease in peak filling rates with exercise but no change in myocardial perfusion. PCr/ATP ratio and peak filling rates correlated strongly at rest and exercise in the HCM group, suggesting that abnormal cardiac energetics in HCM patients is a key mediator of exercise induced diastolic dysfunction commonly seen in this disease [108].

## Comparing Ex-CMR methods

Within this review, all types of available/ previously studied Ex-CMR methodologies have been presented, with each having benefits and weaknesses as displayed in Table 1. To date, treadmill Ex-CMR has demonstrated the most clinical utility, with the multicentre EXACT trial, demonstrating excellent diagnostic value in CAD and superiority over exercise SPECT [45]. Additionally treadmill Ex-CMR is arguably the safest Ex-CMR technique to stress patients with suspected CAD, owing to exercise being performed with 12-lead ECG monitoring which is not feasible with in-scanner methods. Therefore currently, treadmill Ex-CMR is undoubtedly the first choice Ex-CMR method for diagnosing CAD and ischaemia assessment. Studies comparing treadmill Ex-CMR and pharmacological stress CMR, in the form of adenosine/regadenoson stress perfusion or dobutamine stress cine CMR have not been performed. Treadmill Ex-CMR also benefits from simultaneously performing a Bruce protocol treadmill test, which provides additional prognostic and diagnostic information. However as demonstrated in Fig. 2, the average treadmill Ex-CMR test may take longer than pharmacological stress CMR and requires additional specialist equipment and technician training. In-scanner Ex-CMR, as discussed, allows for CMR imaging during multiple stages of continuous exercise. As such, supine bicycle Ex-CMR is best placed for investigating biventricular response and/or flow changes in non-CAD. With further developments, the ability to perform biventricular volume, aortic and pulmonary flow assessment during exercise will allow for accurate direct quantification of aortic and pulmonary artery flow and indirect assessment of mitral regurgitation and tricuspid regurgitation. Given resting CMR quantification of valvular regurgitant flow has demonstrated superior reproducibility and prognostic value over TTE [131–133] and an abnormal response during stress echocardiography can prompt intervention in asymptomatic valve disease [134], Ex-CMR could become an important clinical tool to assess valvular and congenital heart disease. However, commercially available CMR compatible supine cycle ergometers are expensive, therefore institutions wishing to perform Ex-CMR research, in which achieving maximal heart rates are not required, may opt to create a custom device or utilise cheaper alternatives such as prone exercise with ankle weights or resistance bands, indeed isometric hand grip may be preferable for performing exercise CMRS as it produces minimal movement artefact and the modest heart rate increases achieved are sufficient to detect changes in numerous cardiac diseases.

## Future of Ex-CMR

The potential clinical applications for Ex-CMR are considerable, however further technological developments and multicentre trials are needed to demonstrate the clinical utility of Ex-CMR. Ex-CMR will likely dichotomise into treadmill Ex-CMR as an investigation for CAD, and in-scanner Ex-CMR for non-CAD indications, owing to the safer monitoring treadmill Ex-CMR offers and the ability to assess biventricular volumes at numerous exercise stages with in-scanner Ex-CMR. The recently completed multi-centre EXACT-COST trial, if favourable, may assist treadmill Ex-CMR gaining greater clinical use in CAD diagnosis and assessment. In-scanner Ex-CMR could benefit from new faster imaging techniques, to further reduce scanning and exercise time, to take full advantage of the multiparametric benefits CMR offers. Development of imaging techniques which allow volume and flow assessment during continuous exercise that can be analysed in a timely fashion on commercially available software is another important need to increase attainability. 4D flow has recently emerged as a valuable research tool [135]. Its use in Ex-CMR has recently been presented in abstract form demonstrating inefficient right heart function in preterm adults [136]. If feasible and reproducible during continuous exercise it could allow for a comprehensive volumetric and flow assessment during exercise. Clearly further research is needed with such emerging techniques to further enhance the capabilities of Ex-CMR, but with further technological advances Ex-CMR could potentially revolutionise stress CMR.

## Conclusion

Ex-CMR offers the potential to combine the superior imaging quality of CMR with the preferred and physiological method of stress by exercise. Numerous exercise options exist, including CMR scanner adjacent treadmills or in-scanner exercise with a supine cycle ergometer or stepper, prone exercise or IHG exercises. Imaging during maximal intensity in-scanner exercise is feasible using a supine cycle ergometer with ungated real-time imaging. Further advances are required to improve acquisition techniques and decrease scan time, to allow for a comprehensive multi-parametric assessment during exercise, which if feasible could revolutionise stress CMR.

## Supplementary information

**Additional file 2 Video 2.** In-scanner supine cycle ergometer exercise and resulting cine images by un-gated real time cine imaging. Example of the use of the supine cycle ergometer for in-scanner exercise. Video with corresponding cine imaging in the short axis and 4-chamber horizontal long axis from un-gated real time imaging provided by La Gerche et al. [29].

## Data Availability

All literature reviewed is attainable by online search using PubMed or Google Scholar. The only abstract presented in this review is located on the individual journals website.

## Abbreviations

AR: Aortic regurgitation
ATP: Adenosine Triphosphate
BH: Breath hold
BPM: Beats per minute
bSSFP: Balanced steady state free precision
CAD: Coronary artery disease
CC-TGA: Congenitally corrected transposition of the great arteries
CMR: Cardiovascular magnetic resonance
CMRS: Cardiovascular magnetic resonance spectroscopy
CPET: Cardiopulmonary exercise test
CTEPH: Chronic thromboembolic pulmonary hypertension
ECG: Electrocardiogram
EDV: End-diastolic volume
EF: Ejection fraction
EPI: Echo planar imaging
ESV: End systolic volume
Ex-CMR: Exercise cardiovascular magnetic resonance
FB: Free breathing
GRAPPA: Generalized auto-calibrating partially parallel acquisition
HCM: Hypertrophic cardiomyopathy
HRmax: Maximal heart rate
IHG: Isometric hand grip
i-PAH: Idiopathic pulmonary artery hypertension
I-PAT: Integrated parallel imaging
IVC: Inferior vena cava
LPA: Left pulmonary artery
LV: Left ventricle/left ventricular
LVEF: Left ventricular ejection fraction
LVSV: Left ventricular stroke volume
MPS-SPECT: Myocardial perfusion scintigraphy by single photon emission computed tomography
MR-CPET: Magnetic resonance cardiopulmonary exercise testing
MVr: Mitral valve repair
MVR: Mitral valve replacement
NYHA: New York Heart Association
PAH: Pulmonary arterial hypertension
PCMRS: Phosphorus cardiodiovascular magnetic resonance spectroscopy
PCr: Phosphocreatinine
PEA: Pulmonary endartectomy
PPVI: Percutaneous pulmonary valve implantation
RPA: Right pulmonary artery
RV: Right ventricle/right ventricular
RVEF: Right ventricular ejection fraction
RVSV: Right ventricular stroke volume
s-RV: Systemic right ventricle
SENSE: Sensitivity encoding
SV: Stroke volume
SVC: Superior vena cava
T: Tesla
T1DM: Type 1 diabetes mellitus
T2DM: Type 2 diabetes mellitus
TCPC: Total cavo-pulmonary connection
TGA: Transposition of the great arteries
THR: Target heart rate
ToF: Tetralogy of Fallot
TTE: Transthoracic echocardiography
VO2 max: Maximal oxygen consumption
VSD: Ventricular septal defect
